# The Geometry of Concepts: Sparse Autoencoder Feature Structure

**DOI:** 10.3390/e27040344

**Published:** 2025-03-27

**Authors:** Yuxiao Li, Eric J. Michaud, David D. Baek, Joshua Engels, Xiaoqing Sun, Max Tegmark

**Affiliations:** 1 Beneficial AI Foundation (BAIF), Cambridge, MA 02139, USA; yuxiao2234@gmail.com; 2 Department of Physics, Massachusetts Institute of Technology, Cambridge, MA 02139, USA; ericjm@mit.edu (E.J.M.); xqsun@mit.edu (X.S.); 3 Institute for Artificial Intelligence and Fundamental Interaction, Cambridge, MA 02139, USA; dbaek@mit.edu; 4 Department of Electrical Engineering and Computer Science, Massachusetts Institute of Technology, Cambridge, MA 02139, USA; jengels@mit.edu

**Keywords:** sparse coding, mechanistic interpretability, neural networks, large language models, clustering

## Abstract

Sparse autoencoders have recently produced dictionaries of high-dimensional vectors corresponding to the universe of concepts represented by large language models. We find that this concept universe has interesting structure at three levels: (1) The “atomic” small-scale structure contains “crystals” whose faces are parallelograms or trapezoids, generalizing well-known examples such as (*man:woman::king:queen*). We find that the quality of such parallelograms and associated function vectors improves greatly when projecting out global distractor directions such as word length, which is efficiently performed with linear discriminant analysis. (2) The “brain” intermediate-scale structure has significant spatial modularity; for example, math and code features form a “lobe” akin to functional lobes seen in neural fMRI images. We quantify the spatial locality of these lobes with multiple metrics and find that clusters of co-occurring features, at coarse enough scale, also cluster together spatially far more than one would expect if feature geometry were random. (3) The “galaxy”-scale large-scale structure of the feature point cloud is not isotropic, but instead has a power law of eigenvalues with steepest slope in middle layers. We also quantify how the clustering entropy depends on the layer.

## 1. Introduction

While large language models (LLMs) now exhibit a variety of impressive abilities [1,2,3], we largely do not understand the internal cognition that underlies the behavior of these systems. This lack of transparency may pose a challenge for a variety of AI safety [4] concerns. For instance, it may be difficult to tell whether seemingly benign model behavior in any particular instance is sycophantic [5] or deceptive [6] without an analysis of the internals of the system. Such “interpretability” analysis has already shown promise in auditing AI systems [7] to identify misaligned goals [8]. As systems become more powerful, there is a need for methods to further our understanding of the internal representations and algorithms learned by these systems [9,10].

The past year has seen a breakthrough in understanding how large language models work: sparse autoencoders (SAEs) have discovered large numbers of vectors (“features”) in their activation space that can be interpreted as concepts [11,12,13]. These advances build on earlier studies applying sparse coding to artificial neural network representations [14,15,16], and to earlier work in neuroscience on biological neural representations [17,18]. Underlying this work is the idea that neural networks use *sparse coding* to represent concepts in their activation space [19]. In particular, sparse autoencoders are motivated by the assumptions that (1) networks compute a variety of “features” from their input, (2) features are represented as one-dimensional directions in activation space $\left\{d_{i}\right\}$, (3) features are represented simply by adding them to the network’s activations, so activation vectors take the form $\sum_{i}f_{i}d_{i}$, and (4) the coefficients $f_{i}$ are *sparse*—only a small subset of all possible features “fire” at once. The combination of assumptions (2)–(4) has been called the Linear Representation Hypothesis [20,21,22].

If these assumptions hold, we could automatically discover these features with *sparse dictionary learning*. Sparse dictionary learning attempts to learn an overcomplete basis (dictionary) $\left\{d_{i}\right\}$ such that vectors x from a given distribution can be represented as sparse linear combinations of dictionary elements. Sparse autoencoders offer a simple approach to sparse dictionary learning. Sparse autoencoders consist of a learnable encoder function Enc, which maps vectors $x\in R^{n}$ to a hidden latent representation $f\in R^{m}$, and a decoder Dec, which maps latent f back to $\hat{x}\in R^{n}$. The objective of the sparse autoencoder is to accurately reconstruct the input x from a **sparse** latent representation, and they are trained with gradient descent with a loss function like

$$L=||x-{\mathrm{Dec}\left(\mathrm{Enc}\left(x\right)\right)||}_{2}^{2}+{\lambda ||f||}_{0}.$$

Sparse autoencoders use a linear decoder $\mathrm{Dec}\left(f\right)=W_{d}f+b_{d}$, so that the output of the SAE can be interpreted as a linear combination of features: $\hat{x}=\sum_{i}f_{i}W_{d}^{i}+b_{d}$. In practice, hidden latents discovered by sparse autoencoders tend to be more interpretable than neurons, activating in more consistent contexts [11,12], suggesting that they may be learning the true latents underlying the network’s computation. For AI safety, sparse autoencoders have shown some preliminary success: Ref. [7] reports specially training an LLM to have a hidden objective, and then challenging separate teams of researchers to identify this objective. One team was able to quickly identify this objective by looking at sparse autoencoder features that activated when the LLM was prompted to exhibit “potentially concerning behaviors”, and then looking at examples in the training data where that same feature fired.

Although some early work motivating sparse autoencoders suggested that networks would arrange features maximally spread apart (approximately orthogonal) [19], recent works have suggested that features may have a more sophisticated geometric structure [13,22]. Recently, a large collection of SAEs have been made publicly available [23], so it is timely to study their structure at various scales. Thus, the present paper examines sparse autoencoder feature structure at three separate spatial scales, which we refer to informally as the “atom”-scale, “brain”-scale, and “galaxy”-scale. These playful analogies are not meant to be precise, but instead gesture at certain concepts and methods of analysis from other fields which we apply to understanding language model feature structure. We provide project code at https://github.com/ejmichaud/feature-geometry (accessed on 24 March 2025).

This paper is organized as follows. In Section 2, we summarize related work. In Section 3, we investigate if the “atomic” small-scale structure contains “crystals” whose faces are parallelograms or trapezoids, generalizing well-known examples such as (*man:woman::king:queen*). In Section 4, we test if the “brain” intermediate-scale structure has functional modularity akin to biological brains. In Section 5, we study the “galaxy” large-scale structure of the feature point cloud, testing whether it is more interestingly shaped and clustered than an isotropic Gaussian distribution, and conclude in Section 6.

## 2. Related Work

**Neural network geometry**: Many past works have studied the geometry of neural network activations. These works find that the intrinsic dimension of neural network hidden states are much lower than the full model dimension [24], that nearby vectors in activation space are semantically similar [25], and that at local minima well generalizing neural network loss landscapes have many “flat” directions [26]. Other works study how representations evolve through models; one hypotheses is “iterative inference”, which claims that neural networks iteratively refine activations layer by layer [27,28]. A contrasting hypothesis is a circuits view, which holds that information flows in discrete steps along a directed acyclic graph through the model, and representations cleanly change between steps [29]. Another work [30] found that representations of hierarchically related concepts are orthogonal to each other while categorical concepts are represented as polytopes. Our work is in the same vein as these earlier analysis, but differs in an important way because we use the SAE basis, which represents the model’s atomic concept space instead of its activation space.

**SAE feature structure**: Sparse autoencoders (SAEs) are a recent approach for discovering interpretable language model features without supervision, although relatively few works have examined SAE feature structure. Bricken et al. [12] and Templeton et al. [13] both visualize SAE features with UMAP projections and notice that features tend to group together in “neighborhoods” of related features, in contrast to the approximately orthogonal geometry observed in the toy model of Elhage et al. [19]. Engels et al. [22] find examples of SAE structure where multiple SAE features appear to reconstruct a multi-dimensional feature with interesting geometry, and multiple authors have recently speculated that SAE vectors might contain more important structures [31,32]. Bussmann et al. [33] suggest that SAE features are in fact linear combinations of more atomic features, and discover these more atomic latents with “meta SAEs”. Our discussion of crystal structure in SAE features is related to this idea that seemingly atomic representations might be composed of more atomic representations.

**Semantically meaningful linear representations**: Early work found that word embedding methods such as GloVe and Word2vec contained directions encoding semantic concepts, e.g., the well-known formula f(king) − f(man) + f(woman) = f(queen) [34,35,36]. More recent research has found similar evidence of linear representations in sequence models trained only on next token prediction, including Othello board positions [37,38], integer lattices [39], the truth value of assertions [40], and numeric quantities such as longitude, latitude, birth year, and death year [41,42], inspiring the Linear Representation Hypothesis (see above). Recent works have also found causal *function vectors* for in-context learning [43,44,45]. These function vectors induce the model to perform a certain task when added into the model’s hidden states. Our discussion of crystal structures builds upon these previous works by finding these task vectors and parallelogram structures in sparse autoencoder representations.

## 3. “Atom”-Scale: Crystal Structure

**Figure 1:**
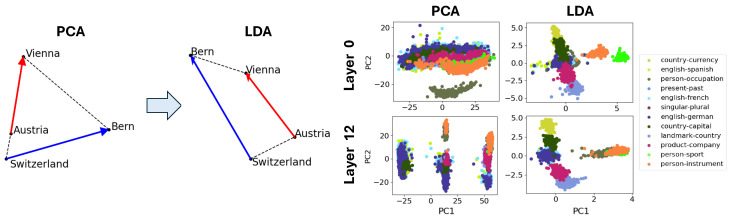
Parallelogram and trapezoid structure is revealed (**left**) when distractor dimensions were projected out from the activations using LDA. LDA results in tighter clusters of pairwise Gemma-2-2b activation differences (**right**), where each cluster corresponds to a different semantic transformation. Distractor features are defined as those that are not related to semantics of the text; for instance, the first principal component of Gemma-2-2b’s Layer 0 activations (top left figure on the right panel) represents word length. Parallelogram or trapezoid structures suggest that there is a unique direction in the activation space that represents each semantic transformation.

In this section, we search for what we term *crystal* structure in the point cloud of SAE features. By this we mean geometric structure reflecting semantic relations between concepts, generalizing the classic example of (a,b,c,d) = (man,woman,king,queen) forming an approximate *parallelogram* where b−a≈d−c. This can be interpreted in terms of two *function vectors* b−a and c−a that turn male entities female and turn entities royal, respectively. We also search for *trapezoids* with only one pair of parallel edges b−a∝d−c (corresponding to only one function vector); Figure 1 (right) shows such an example with (a,b,c,d)=(Austria,Vienna,Switzerland,Bern), where the function vector can be interpreted as mapping countries to their capitals. Studying these crystal structures is important because they provide insight into how LLMs internally represent semantic operations and relational knowledge. For instance, function vectors help us assess the extent to which semantic structures within models align with human intuition and language logic, as explored in the recent literature [43,46].

We search for crystals by computing all pairwise difference vectors and clustering them using the K-means algorithm [47], where the vectors could be either the original model’s hidden state activations (*model crystal*) or SAE features’ decoder vectors (*SAE crystal*). We use Gemma-2-2b for the experiment. If there is a direction that represents each semantic transformation, we expect each resulting cluster to correspond to each function vector. In other words, any pair of difference vectors in a cluster will form a trapezoid or parallelogram, depending on whether the difference vectors are normalized or not before clustering (or, equivalently, whether we quantify similarity between two difference vectors via Euclidean distance or cosine similarity).

Our initial search for SAE crystals found mostly noise. To investigate why, we decided to focus on activations of the model’s early layer, where many SAE features correspond to a single token. Since SAE feature vectors in the early layers are often closely related to the corresponding model activations, we believed that studying the activations of these early layers could help clarify why our initial crystal search primarily found noise. Therefore, we studied Gemma-2-2b residual stream activations for previously reported word ↦ word function vectors from the dataset of [43]. Figure 1 illustrates that candidate crystal quadruplets are typically far from being parallelograms or trapezoids. This is consistent with multiple papers pointing out that (man,woman,king,queen) is not an accurate parallelogram either.

**Figure A2:**
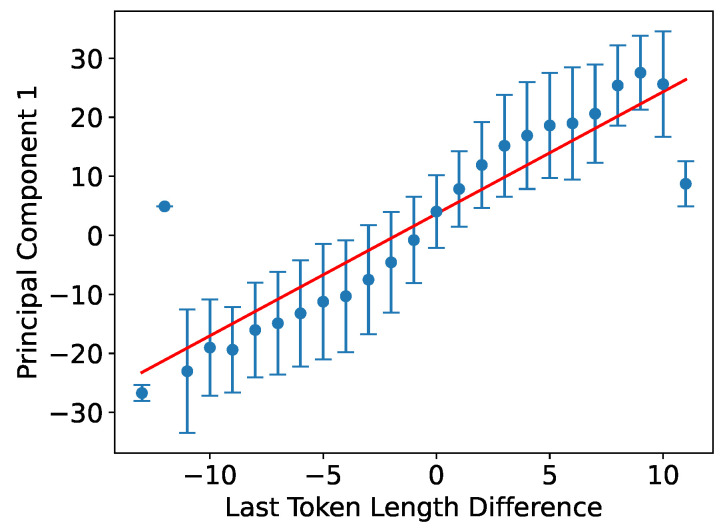
Plot of the first principal component in the difference space as a function of last token length difference in Gemma-2-2b Layer 0. The linear relationship indicates that the first principal component encodes the length difference between two words’ last tokens.

We believe the reason to be the presence of what we term *distractor features*. We define distractor features to be the features that are not related to semantics of the text. For example, we find that the horizontal axis in Figure 1 (right) corresponds mainly to word length (Appendix B, Figure A2), which is semantically irrelevant and wreaks havoc on the trapezoid (left), since “Switzerland” is much longer than the other words. However, these distractor features were not always interpretable; in some cases, it was difficult to associate features with any clear linguistic property.

To eliminate such semantically irrelevant distractor vectors, we wish to project the data onto a lower-dimensional subspace orthogonal to them. For the [43] dataset, we accomplish this with linear discriminant analysis (LDA) [48], which projects onto signal-to-noise eigenmodes where “signal” and “noise” are defined as the covariance matrices of inter-cluster variation and intra-cluster variation, respectively. Figure 1 illustrates that this dramatically improves the cluster and trapezoid/parallelogram quality, highlighting that distractor features can hide existing crystals.

## 4. “Brain”-Scale: Meso-Scale Modular Structure

We now zoom out and look for larger-scale structure. In particular, we investigate if *functionally* similar groups of SAE features (which tend to fire together) are also *geometrically* similar, forming “lobes” in the activation space. We refer to this analyis as “brain”-scale because, in animal brains, functionally similar groups of neurons also typically cluster together spatially. For example, Broca’s area is involved in speech production, the auditory cortex processes sound, and the amygdala is primarily associated with processing emotions. We are curious whether we can find analogous functional modularity in the SAE feature space. While prior work has qualitatively observed that semantically related features are spatially close via UMAP projections of features [12,13], we aim to more precisely quantify the relationship between functional similarity and spatial similarity.

We test a variety of methods for automatically discovering such functional “lobes” and for quantifying if they are spatially modular. We define a **lobe partition** as a partition of the SAE feature point cloud into *k* subsets (“lobes”) that are computed *without positional information*. Instead, we identify such lobes based on them being *functionally* related, specifically, tending to fire together within a document.

To automatically identify functional lobes, we first compute a histogram of SAE feature co-occurrences. We take Gemma-2-2b and pass documents from The Pile [49] through it. In this section, we report results with a Layer 12 residual stream SAE with 16k features and an average L0 of 41. For this SAE, we record the features that fire (we count a feature *i* as firing if its encoder assigns it a coefficient $f_{i}>1$). Features are counted as co-occurring if they both fire within the same block of 256 tokens—this length provides a coarse “time resolution” allowing us to find tokens that tend to fire together within the same document rather than just at the same token. We use a max context length of 1024, and only use one such context per document, giving us at most four blocks (and histogram updates) per document of The Pile. We compute histograms across 50k documents. Given this histogram, we compute an affinity score between each pair of SAE features based on their co-occurrence statistics and perform spectral clustering on the resulting affinity matrix. We use the spectral clustering implementation of scikit-learn [50] with default settings with varying choice of n_clusters.

**Figure 2:**
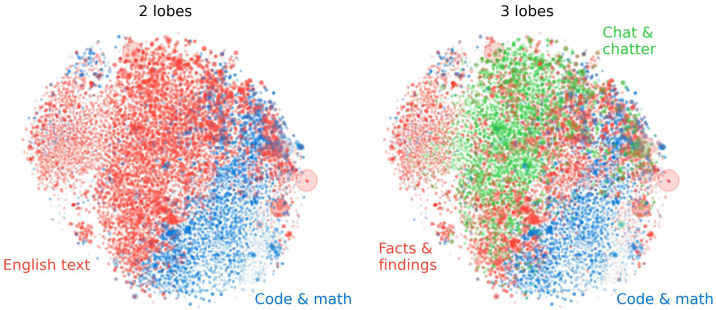
Features in the SAE feature point cloud identified that tend to fire together within documents are seen to also be geometrically co-located in functional “lobes”, here down-projected to 2D with t-SNE with point size proportional to feature frequency. A 2-lobe partition (**left**) is seen to break the point cloud into roughly equal parts, active on code/math documents and English language documents, respectively. A 3-lobe partition (**right**) is seen to mainly subdivide the English lobe into a part for short messages and dialogue (e.g., chat rooms and parliament proceedings) and one primarily containing long-form scientific papers.

In Figure 2, we visualize lobes discovered with this method with n_clusters=2, 3 via a t-SNE projection [51]. For this figure, we used the “phi coefficent” as the measure of co-occurrence similarity between features. We find that lobes visually appear to be spatially localized. For instance, features which fire primarily on math and code documents tend to cluster together spatially.

**Figure 3:**
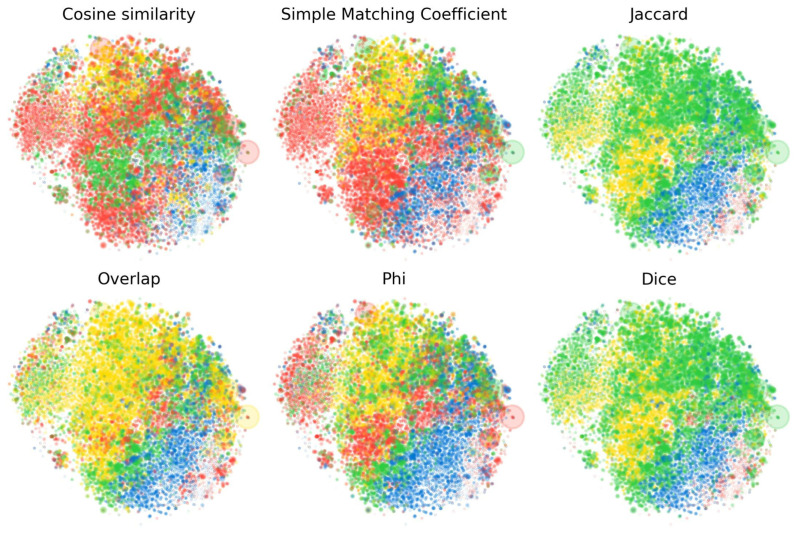
Comparison of the lobe partitions of the SAE point cloud discovered with different affinity measures, with the same t-SNE projection as Figure 2. In the top left, we show clusters computed from **geometry**, the cosine similarity between features as the affinity score for spectral clustering. All other measures are based on whether SAE features co-occur (fire together) within 256-token blocks, using different measures of affinity. Although the phi coefficient predicts spatial structure best, all co-occurrence measures are seen to discover the code/math lobe.

We experiment with the following notions of co-occurrence-based affinity: simple matching coefficient, Jaccard similarity, Dice coefficient, overlap coefficient, and phi coefficient, which can all be computed just from a co-occurrence histogram. In the Section Co-Occurrence Measures, we review definitions for each of these and in Figure 3 illustrate how the choice between them affects the resulting lobe t-SNE plots. We also show how lobes appear when we cluster based on geometry directly using cosine similarities, as described below.

While these plots show a qualitative relationship between co-occurrence and feature geometry, we aim to quantify this relationship. Our null hypothesis is that functionally similar points (of commonly co-occurring SAE features) are uniformly distributed throughout the activation space, showing no spatial modularity. To quantify how statistically significant this is, we use two approaches to rule out the null hypothesis:

1. While we can cluster features based on whether they co-occur, we can also perform spectral clustering based on the cosine similarity between SAE feature decoder vectors. So instead of feature affinity values being, e.g., their co-occurrence phi coefficient, affinity matrix values are instead computed simply from feature geometry as $A_{ij}=d_{i}\cdot d_{j}$. Given a clustering of SAE features using cosine similarity and a clustering using co-occurrence, we compute the mutual information between these two sets of labels. In some sense, this measures the amount of information about geometric structure that one obtains from knowing functional structure. We report the adjusted mutual information [52] as implemented by scikit-learn [50], which corrects for chance agreements between the clusters.
2. Another conceptually simple approach is to train models to predict which functional lobe a feature is in from its geometry. To accomplish this, we take a given set of lobe labels from our co-occurrence-based clustering, and train a logistic regression model to predict these labels directly from the point positions, using an 80-20 train–test split and reporting the balanced test accuracy of this classifier.

**Figure 4:**
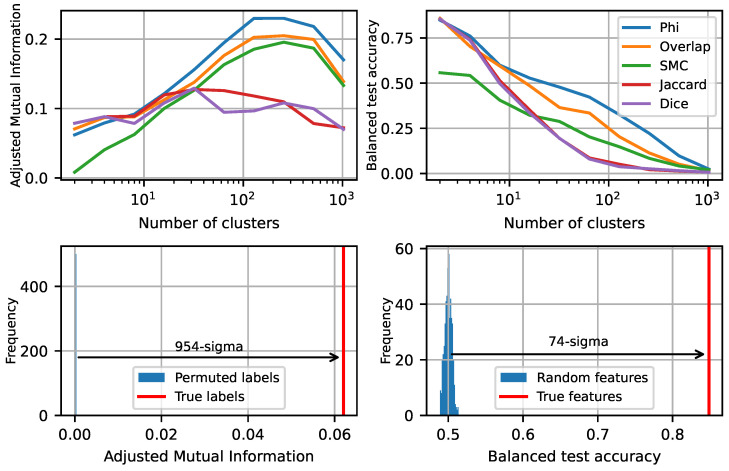
(**top left**): Adjusted mutual information between spatial clusters and functional (co-occurrence-based) clusters. (**top right**): logistic regression balanced test accuracy, predicting co-occurrence-based cluster label from position. (**bottom left**): Adjusted mutual information with randomly permuted cosine similarity-based clustering labels. (**bottom right**): balanced test accuracy with random unit-norm feature vectors. The statistical significance reported is for phi-based clustering into lobes.

Figure 4 shows that for both measures, the phi coefficient gives the best correspondence between functional lobes and feature geometry. To show that this is statistically significant, we randomly permute the cluster labels from the cosine similarity-based clustering and measure the adjusted mutual information. We also re-initialize the SAE feature decoder directions from a random Gaussian and normalize, and then train logistic regression models to predict functional lobe from these random feature directions. Figure 4 (bottom) shows that both tests rule out the null hypothesis with high significance, at 954 and 74 standard deviations, respectively, clearly demonstrating that the lobes we see are real and not a statistical fluke.

**Figure 5:**
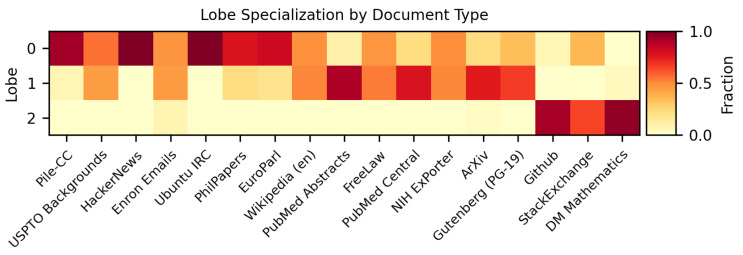
Fraction of contexts in which each lobe had the highest proportion of activating features. For each document type, these fractions sum to 1 across the lobes. We see that lobe 2 typically disproportionately activates on code and math documents. Lobe 0 and 1 activate on other documents, with lobe 0 activating more on documents containing short text and dialogue (chat comments, parliamentary proceedings) and lobe 1 activating more on scientific papers.

To assess what each lobe specializes in, we run 10k documents from The Pile through Gemma-2-2b, and again record which SAE features at Layer 12 fire within blocks of 256 tokens. For each block of tokens, we record which lobe has the highest proportion of its features firing. Each document in The Pile is attached with a name specifying the subset of the corpus that document is from. For each document type, for each 256-token block within a document of that type, we record which lobe had the highest proportion of its SAE features firing. Across thousands of documents, we can then look at a histogram of which lobes were maximally activating across each document type. We show these results for three lobes, computed with the phi coefficient as the co-occurrence measure, in Figure 5. This forms the basis for our lobe labeling in Figure 2.

These findings raise interesting questions about whether individual sparse autoencoder features are the most natural units for understanding neural networks [53,54]. In biological brains, one can study individual neurons, groups of neurons, groups of groups of neurons, and so on up to very large-scale structures, and it is not clear a priori what “scale” of analysis will be most fruitful [55]. We may face a similar ambiguity with sparse autoencoder features, since, as we have seen, groups of co-occurring, geometrically related features can be interpretable and studied in their own right. This question, of whether there is a right “scale” of analysis for SAE features, is made even more salient by the observation in prior work of “feature splitting” [12].

## 5. “Galaxy”-Scale: Large-Scale Point Cloud Structure

In this section, we further broaden our perspective to analyze the “galaxy”-scale structure of the point cloud, focusing on its overall shape and clustering properties. This analysis is loosely inspired by work in astronomy [56] characterizing the shape [57] and substructure [58] of galaxies. We start by formulating a simple null hypothesis: *The point cloud is drawn from an isotropic multivariate Gaussian distribution.*

**Figure 6:**
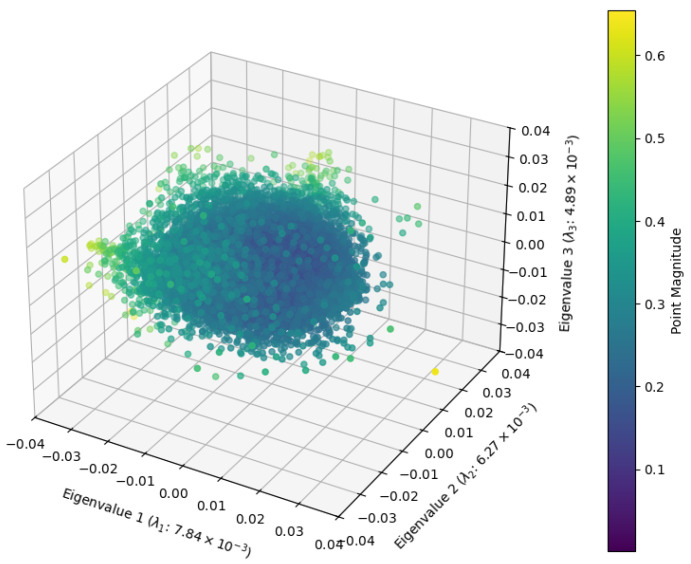
Three-dimensional point cloud visualizations of top PCA components for the Gemma-2-2b Layer 12 SAE features.

To test this, we analyze the covariance of the data. As illustrated in Figure 6, the eigenvalue spectrum deviates from isotropy, meaning the cloud exhibits directional structure rather than being purely spherical. Even within the first three principlal components, the point cloud is anisotrophic, with some principal axes slightly wider than others.

To quantify these deviations, we analyze the eigenvalue spectrum of the covariance matrix, comparing it to theoretical expectations from random matrix theory (RMT).

### 5.1. Shape Analysis

In RMT, the covariance matrix of *N* random vectors from a multivariate Gaussian distribution follow a Wishart distribution [59]. Under this assumption, we would expect the eigenvalues to be relatively uniform or to follow the Marcenko–Pastur law [60]. In contrast, we observe a surprising derivation:

- The eigenvalue spectrum of the point cloud decays as a power law rather than following the expected Wishart behavior.
- As shown in Figure 6, this power law decay is more pronounced in SAE features compared to raw activations.

Since the abrupt drop off seen for the smallest eigenvalues is caused by limited data and vanishes in the limit N→∞, we dimensionally reduce the point cloud to its 100 largest principal components for all subsequent analysis in this section. We describe the shape of this high-dimensional point cloud as resembling a “fractal cucumber”, whose width in successive dimensions falls off like a power law. We find such power law scaling is significantly less prominent for activations than for SAE features; it will be interesting for further work to investigate its origins.

**Figure 7:**
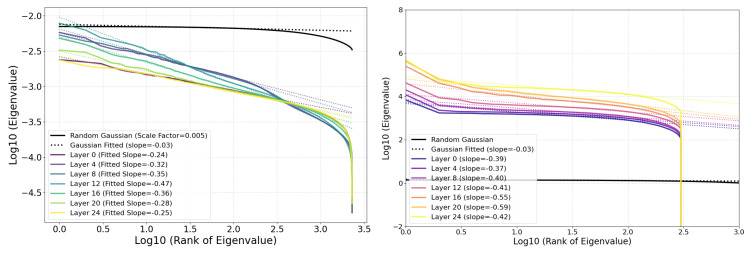
Eigenvalue distributions for SAE features and activations. Eigenvalues of the covariance matrix for SAE features (**left**) and neural activations (**right**) decay approximately as a power law, with slopes varying across layers. A scaled isotropic Gaussian spectrum is shown for comparison, highlighting the significantly steeper decay for SAE features. Eigenvalue spectra for activations show a much slower decay compared to SAE features, indicating weaker power law behavior and distinct geometric structures.

**Figure 8:**
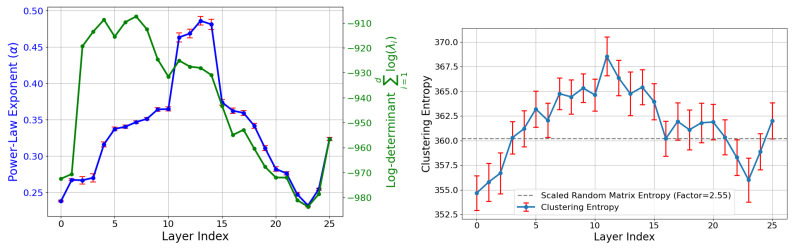
Layer-wise analysis of latent representations. (**left**): The power law slope (α) of the eigenvalue spectrum (blue) and the log-determinant of the covariance matrix (green) vary across layers. Both metrics peak in intermediate layers, indicating significant structural changes in the latent space. (**right**): Estimated clustering entropy across layers with 95% confidence intervals. Middle layers exhibit reduced clustering entropy, while earlier and later layers show higher entropy, reflecting distributed and concentrated feature representations, respectively.

Figure 7 (left) shows how the slope of the aforementioned power law depends on LLM layer, computed via linear regression against the 100 largest eigenvalues. We see a clear pattern where middle layers have the steepest power law slopes: (Layer 12 has slope −0.47, while early and late layers (e.g., Layers 0 and 24) have shallower slopes (−0.24 and −0.25), respectively. This may hint that middle layers act as a bottleneck, compressing information into fewer principal components, perhaps optimizing for more efficient representation of high-level abstractions. Figure 7 (right) compares the eigenvalue spectra of SAE features and neural activations, indicating a significantly steeper power law decay for SAE features. Activations, in contrast, exhibit a much slower decay, indicating weaker power law behavior and distinct geometric structures in the latent space. Figure 8 (left) explores the effective cloud volume (the determinant of the covariance matrix) of the point cloud, quantified by the log-determinant of the covariance matrix across layer. This volume variation further reflects the layer-specific changes in the structure and complexity of the latent space.

### 5.2. Clustering Analysis

Clustering of galaxies or microscopic particles is often quantified in terms of a power spectrum or correlation function. This is complicated for our very high-dimensional data, since the underlying density varies with radius and, for a high-dimensional Gaussian distribution, is strongly concentrated around a relatively thin spherical shell. For this reason, we instead quantify clustering by estimating the *entropy* of the distribution that the point cloud is assumed to be sampled from. We estimate the entropy *H* from our SAE feature point cloud using the *k*-th nearest neighbor (k-NN) method [61,62], computed as follows,

(1) $$H_{features}=\frac{d}{n}\sum_{i=1}^{n}\log\left(r_{i}+\theta \right)+\log\left(n-1\right)-\Psi$$

where $r_{i}$ is the distance to the *k*-th nearest neighbor for point *i*, and *d* is the dimensionality of the point cloud; *n* is the number of points; the constant Ψ is the digamma term from the k-NN estimation. As a baseline, the Gaussian entropy represents the maximum possible entropy for a given covariance matrix. For a Gaussian distribution with the same covariance matrix, the entropy is computed as follows:

(2) $$H_{gauss}=\frac{d}{2}\left(1+\log(2\pi )\right)+\sum_{i=1}^{d}\log\left(\lambda_{i}\right)$$

where $\lambda_{i}$ are the eigenvalues of the covariance matrix. We define the **clustering entropy** (often referred to as “negentropy” in physics as $H_{gauss}-H$, i.e., how much lower the entropy is than its maximum allowed value).

The estimated clustering entropy is shown in Figure 8 (right), plotted across different layers. The results indicate that the SAE point cloud is strongly clustered, particulary in the middle layers. This observation aligns with the reduced clustering entropy seen at intermediate layers, suggesting significant structural differences in the latent representations.

In future work, it will be interesting to investigate whether these variations depend mainly on the prominence of crystals or lobes in different layers, or have an altogether different origin (entirely different underlying mechanisms).

## 6. Conclusions

We have searched for structure in the SAE concept universe at three levels: (1) The “atomic” small-scale structure contains “crystals” whose faces are parallelograms or trapezoids, generalizing well-known examples such as (*man:woman::king:queen*), may be revealed when projecting out semantically irrelevant distractor features. (2) The “brain” intermediate-scale structure has significant spatial modularity; for example, math and code features form a “lobe” akin to functional lobes seen in neural fMRI images. (3) The “galaxy” large-scale structure of the feature point cloud is not isotropic, but instead has a power law of eigenvalues with steepest slope in middle layers.

While we have observed that SAE features exhibit geometric structure at multiple scales, we have not explained *why* this structure forms. We think that further work that not only studies the structure of SAE features, but also seeks to explain the origin of this structure, could be highly valuable. Such work may lead to refinements to our theory of how networks represent features in superposition or to insights that improve sparse autoencoder performance.

We hope that our findings serve as a stepping stone toward deeper understanding of SAE features and the workings of large language models, and that this deeper understanding will eventually help to improve the safety of AI systems as they continue to grow in power.

## Figures and Tables

**Figure A1:**
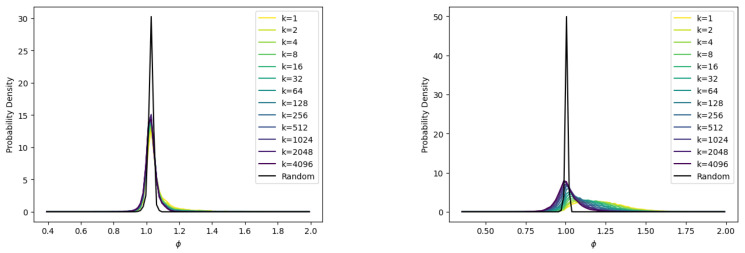
Histogram, over all features, of phi coefficient with *k*-th nearest cosine similarity neighbor for (**left**) Layer 0 and (**right**) Layer 12. Each line represents a different *k*. The “random” line is plotted by drawing a random feature for each feature, then computing the phi coefficient. Features with higher cosine similarity have higher phi coefficients, but this is less pronounced in Layer 0 compared to Layer 12.

**Figure A3:**
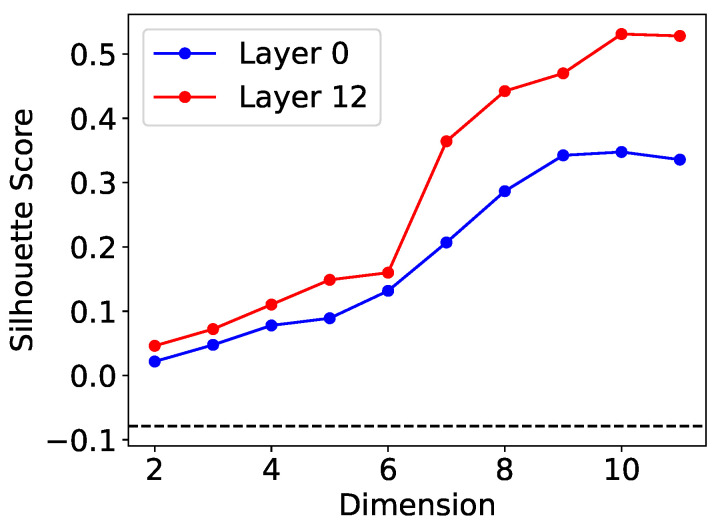
Silhouette score, a measure of clustering quality, as a function of reduced dimension in LDA. The plot indicates that training an affine transformation for semantic cluster separation is easier in middle layers (Layer 12), where the model starts to develop concept-level understanding of the input.

**Figure A4:**
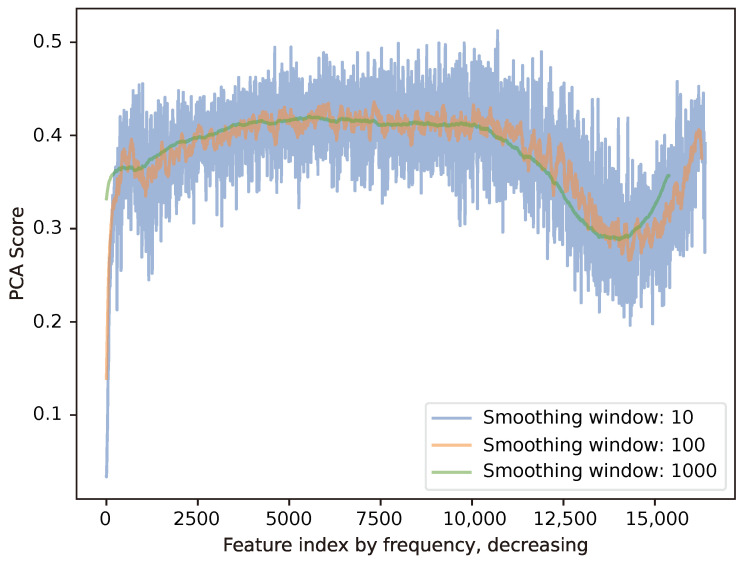
Smoothed PCA scores for each SAE feature of Layer 12, width 16k, $L_{0}=176$ Gemma Scope 2b SAE, sorted by frequency. PCA score = $\frac{1}{n}\sum_{i}i*{\left(pca_{i}@feature_{j}\right)}^{2}$, where *n* is the number of PCA features. The smoothed curves just average this somewhat noisy metric over adjacent sorted features. This measures approximately where in the PCA each SAE feature lies, and shows that there is a dip into earlier PCA features in the last third of SAE features.

## Data Availability

We provide code to replicate our results at this repository: https://github.com/ejmichaud/feature-geometry (accessed on 24 March 2025).

## Appendix A. Additional Information on Brain Lobes

### Co-Occurrence Measures

Definitions of co-occurrence-based affinity measures: Let $n_{ij}$ be the number of times features *i* and *j* co-occur. Let $m_{11}$ be number of times *i* and *j* co-occur, $m_{00}$ be number of times *i* and *j* both do not occur, $m_{10}$ be number of times *i* occurs but *j* does not, $m_{1•}$ be number of times *i* occurs and *j* either occurs or not, and so on. Then, the following can be determined.

Jaccard similarity, Ref. [63], is as follows:

$$J_{ij}=\frac{\left|i\cap j\right|}{\left|i\cup j\right|}=\frac{n_{ij}}{n_{ii}+n_{jj}-n_{ij}}$$

Dice score, Ref. [64],is as follows:

$$DSC_{ij}=\frac{2|i\cap j|}{\left|i|+|j\right|}=\frac{2n_{ij}}{n_{ii}+n_{jj}}$$

The overlap coefficient is as follows:

$$overlap_{ij}=\frac{\left|i\cap j\right|}{\min\left(|i|,|j|\right)}=\frac{n_{ij}}{\min\left(n_{ii},n_{jj}\right)}$$

The simple matching coefficient is

$$SMC_{ij}=\frac{m_{00}+m_{11}}{m_{00}+m_{11}+m_{01}+m_{10}}$$

The phi coefficient, Ref. [65], is

$$\phi_{ij}=\frac{m_{11}m_{00}-m_{10}m_{01}}{\sqrt{m_{1•}m_{0•}m_{•1}m_{•0}}}$$

## Appendix B. Understanding Principal Components in Difference Space

Figure A2 shows that the first principal component encodes mainly the length difference between two words’ last tokens in Gemma-2-2b Layer 0.

## Appendix C. Breaking Down SAE Vectors by PCA Component

An additional investigation of structure we undertake is quantifying how SAE vectors are distributed throughout the PCA components of the activations vectors. To accomplish this, we define a PCA score:

$$\mathrm{PCA score}\left(feature_{j}\right)=\frac{1}{n}\sum_{i}i*{\left(pca_{i}@feature_{j}\right)}^{2}$$

This metric is a weighted sum between 0 and 1 measuring approximately where in the PCA each SAE feature lies. In Figure A4, we plot this metric on a single Gemma Scope SAE (the results look similar on all Gemma Scope SAEs), and we see that there is an intriguing dip into earlier PCA features in the last third of SAE features.
